# Adjunctive Immersive Virtual Reality for Urinary Incontinence-Related Quality of Life in Postmenopausal Women with Urinary Incontinence: A Non-Randomized Controlled Pilot Trial

**DOI:** 10.3390/jcm15197726

**Published:** 2026-10-07

**Authors:** Gabriela Kołodyńska, Błażej Cieślik, Aleksandra Piątek, Maciej Zalewski, Joanna Szczepańska-Gieracha, Waldemar Andrzejewski

**Affiliations:** 1 Department of Physiotherapy, Wroclaw University of Health and Sport Sciences, 51-612 Wroclaw, Poland; gabriela.kolodynska@awf.wroc.pl (G.K.);; 2 Healthcare Innovation Technology Lab, IRCCS San Camillo Hospital, 30126 Venice, Italy; 3 Department of Gynaecology, Independent Public Health Care Center of the Ministry of the Interior and Administration in Wroclaw, 50-233 Wroclaw, Poland; 4 Institute of Health, DSW Ideis University, 53-611 Wroclaw, Poland

**Keywords:** urinary incontinence, virtual reality, electrostimulation, electromyographic biofeedback, pelvic floor rehabilitation, quality of life

## Abstract

**Background/Objectives:** This study examined whether adding relaxation-oriented immersive virtual reality (VR) to pelvic floor muscle electrostimulation with electromyographic (EMG) biofeedback was associated with additional improvement in incontinence-related quality of life among postmenopausal women. **Methods:** In this exploratory non-randomized controlled pilot trial, 40 postmenopausal women received electrostimulation with EMG biofeedback alone (ES; *n* = 20) or the same intervention preceded by VR (VR + ES; *n* = 20). Participants completed eight sessions over 2 weeks and were assessed at baseline (T0), post-intervention (T1), and 2-week follow-up (T2); T1 was the prespecified primary assessment. The primary outcome was the Incontinence Quality of Life Questionnaire (I-QOL) total score. Secondary outcomes included pelvic floor EMG, bladder-diary variables, other quality-of-life measures, perceived stress, and self-esteem. Baseline-adjusted linear mixed models were used. The registry record was submitted before enrollment (NCT07070063). **Results:** Thirty-six participants contributed to the primary analysis. The adjusted between-group difference in I-QOL was −1.65 points at T1 (95% CI, −10.03 to 6.74; *p* = 0.70) and −4.00 points at T2 (95% CI, −12.38 to 4.39; *p* = 0.35). Adjusted contrasts were non-significant for the remaining questionnaire and EMG outcomes. Selected bladder-diary outcomes differed between groups, but these analyses were exploratory, unadjusted for multiple comparisons, and did not directly quantify urinary leakage. **Conclusions:** The study did not detect a statistically significant additional improvement in incontinence-related quality of life with adjunctive VR. Confidence intervals remained compatible with potentially meaningful effects in either direction. Given the small sample and non-randomized design, the findings are preliminary and hypothesis-generating.

## 1. Introduction

Urinary incontinence (UI) is one of the most common chronic conditions affecting women worldwide [1]. The latest results of a meta-analysis conducted by Rostami et al. indicate that urinary incontinence affects up to 27.4% of adult women in developing countries [2]. The frequency of urinary incontinence rises progressively with advancing age and has been estimated to occur in approximately 58–84% of older women [3]. Reported prevalence rates differ considerably across studies, largely due to variations in diagnostic definitions and measurement methods. In women aged over 60 years, overall prevalence has been reported to range from 38% to 55% [4]. Urinary incontinence is associated with marked deterioration in quality of life, affecting emotional well-being, interpersonal relationships, occupational functioning, and participation in daily activities. Many affected women modify their lifestyle to avoid leakage episodes, which may lead to social isolation, reduced physical activity, and worsening psychological distress [5]. Beyond physical symptoms, UI is increasingly recognized as a condition strongly associated with mental health problems. Women with UI symptoms often report embarrassment, loss of self-confidence, anxiety related to unpleasant odor or leakage in public, and depressive symptoms resulting from reduced independence and chronic symptom burden [6,7]. Previous studies have shown bidirectional associations between psychological distress and urinary symptoms in women [8]. This bidirectional relationship suggests that effective management of UI should address both pelvic floor dysfunction and the psychological consequences of the disorder. These associations highlight psychological well-being as an important component of the overall burden of UI [9,10].

Conservative treatment remains the first-line therapeutic approach for many women with urinary incontinence and commonly includes pelvic floor muscle training, behavioral interventions, biofeedback and electrostimulation [11,12]. However, adherence to pelvic floor muscle training can be challenging and may be influenced by motivational and contextual factors [13]. Therefore, there is growing interest in supportive interventions that may improve both therapeutic engagement and mental well-being during rehabilitation.

Virtual reality (VR) has increasingly been used in rehabilitation to provide immersive and interactive therapeutic environments. Previous studies have shown that VR can be used to support rehabilitation and may enhance motivation and engagement during treatment [14,15]. However, evidence regarding the potential role of VR in women with urinary incontinence remains limited [16]. In this context, VR may serve as an adjunctive intervention aimed at supporting relaxation and engagement during pelvic floor rehabilitation. By reducing treatment-related stress and increasing engagement, such an intervention could facilitate participation in rehabilitation and potentially improve patient-reported quality of life. Whether adding VR to conventional pelvic floor rehabilitation translates into measurable benefits in incontinence-related quality of life, urinary symptoms, or psychological outcomes remains unclear.

The aim of this study was to examine whether adding virtual reality to pelvic floor muscle electrostimulation with EMG biofeedback was associated with greater improvement in incontinence-related quality of life, while also exploring effects on EMG parameters, micturition outcomes, symptom burden, condition-specific quality of life, and psychological outcomes.

## 2. Materials and Methods

### 2.1. Study Design and Registration

This study was initially planned as a randomized controlled trial and was submitted for registration at ClinicalTrials.gov on 7 July 2025 (NCT07070063), before enrollment of the first participant on 15 July 2025, and was first posted on 17 July 2025. The initial submission specified change from baseline in the I-QOL total score at the end of the 2-week treatment period as the primary outcome; this corresponds to T1 in the present manuscript. T2 was an additional 2-week post-treatment follow-up that was not included in the initial registration. Before enrollment, the allocation procedure was changed from randomized to non-randomized because the VR system was available for a limited loan period. Participants were subsequently allocated sequentially according to VR-system availability.

The ClinicalTrials.gov record was updated after completion of recruitment and follow-up, with the revised update submitted on 22 July 2026, to reflect the study as conducted. The intervention procedures remained unchanged; the update clarified that VR and ES/EMG biofeedback were delivered using separate devices and that the VR session preceded ES/EMG biofeedback. The revised secondary outcomes and the additional T2 assessment are treated as exploratory. Material differences between the original registration and the study as conducted are summarized in Supplementary Table S1. No detailed statistical analysis plan was prospectively registered.

The resulting design was an assessor-blinded, two-arm, parallel-group non-randomized controlled pilot trial. Participants received either pelvic floor muscle electrostimulation with EMG biofeedback alone (ES) or the same intervention preceded by an immersive virtual reality session (VR + ES). The study is reported in accordance with the Transparent Reporting of Evaluations with Nonrandomized Designs (TREND) statement.

### 2.2. Study Setting

The study was conducted at the Independent Public Health Care Center of the Ministry of the Interior and Administration in Wroclaw, Poland. Participant recruitment, intervention delivery, and data collection were conducted between July 2025 and February 2026. The intervention period lasted 2 weeks and comprised eight treatment sessions administered four times per week. Outcome assessments were performed at baseline (T0), immediately after completion of the intervention (T1), and at the 2-week follow-up (T2). The study was approved by the Bioethics Committee of Wroclaw Medical University, Poland (approval no. 49/2025), and all participants provided written informed consent before enrollment.

### 2.3. Sample Size

The target sample size was set a priori at 40 participants, with 20 participants per group. This number was based on recruitment feasibility and general methodological recommendations for pilot studies. Previous methodological work has suggested approximately 12–15 participants per group as a reasonable minimum for pilot studies [17,18]. To account for potential attrition, the target was increased to 20 participants per group. No formal a priori power calculation was performed.

### 2.4. Participants

In total, 52 women were screened for eligibility, of whom 40 fulfilled the inclusion criteria and were enrolled in the study. Eligible participants were postmenopausal women with urinary incontinence who met the clinical eligibility criteria used at recruitment. UI was classified as stress urinary incontinence (SUI), urgency urinary incontinence (UUI), or mixed urinary incontinence (MUI) according to standard clinical definitions [19]. The diagnosis and UI subtype were established by a gynecologist primarily on the basis of medical history and gynecological examination; in cases of diagnostic uncertainty, bladder-diary data and/or urodynamic testing were additionally considered. The Gaudenz questionnaire was used as part of the clinical characterization of UI. Clinical eligibility was based on the gynecologist’s overall assessment of symptom presentation and diagnostic findings; no separate severity grade was analyzed as an outcome in the present study. Baseline symptom burden is therefore described using UI subtype and baseline I-QOL scores.

Women were excluded if they had significant neurological conditions affecting bladder function, were concurrently participating in other therapeutic interventions, were pregnant, or were unable to provide informed consent.

The final study sample consisted of 40 women assigned to either the electrostimulation group (ES) or the virtual reality–assisted electrostimulation group (VR + ES), with 20 participants in each group. In the ES group, 5 participants (25.0%) had SUI, 1 (5.0%) had UUI, and 14 (70.0%) had MUI. In the VR + ES group, 2 participants (10.0%) had SUI, 3 (15.0%) had UUI, and 15 (75.0%) had MUI. This distribution is also presented in Table 1.

### 2.5. Group Allocation

Participants were assigned to the ES or VR + ES group according to the organizational and technical availability of the VR system during the recruitment period. Participants were recruited sequentially. The VR + ES group was recruited first, during the limited period in which the VR system was on loan and available at the study center. After the VR equipment had been returned, recruitment continued for the ES group because the electrostimulation and EMG biofeedback equipment remained routinely available. Assignment was therefore determined solely by the recruitment period in relation to VR availability. The recruitment periods did not overlap, and no individual participant-level allocation decisions were made. Participant preference, UI severity, or other clinical characteristics did not influence assignment. No random allocation sequence was generated, and allocation concealment was not used. All participants were recruited from the same clinical setting, and the same eligibility criteria were applied in both groups. Both groups were treated by the same physiotherapist and core study staff, who used the same electrostimulation and EMG biofeedback procedures.

### 2.6. Interventions

All participants underwent a 2-week intervention program consisting of eight treatment sessions. Electrostimulation of the pelvic floor muscles was performed in both groups with the patient in a supine position, with the lower limbs supported on wedges. Vaginal electrodes were used to deliver stimulation and to record muscle activity. Electrostimulation and EMG biofeedback were delivered using a NeuroTrac^®^ MyoPlus 4 Pro device (model MYO420P; Verity Medical Ltd., Braishfield, Hampshire, UK) with NeuroTrac^®^ PC software (version 5.0). Stimulation parameters were selected based on those reported in the literature as effective in the treatment of stress urinary incontinence. The same standardized stimulation protocol was applied across UI subtypes to ensure consistency. Because MUI includes a stress component, the protocol was clinically relevant to most of the sample; however, evidence supporting these specific parameters is stronger for SUI than for UUI. Neuromuscular electrical stimulation was administered using an individually assigned intravaginal probe (EveryWay Medical Instruments Co., Ltd., PR-02, New Taipei City, Taiwan). Symmetrical biphasic rectangular pulses were delivered at a frequency of 50 Hz and a pulse width of 250 μs. The duty cycle consisted of 5 s of stimulation followed by 10 s of rest, with 1-s ramp-up and ramp-down times. Each session lasted 20 min, and each participant completed eight treatment sessions. Stimulation intensity was individualized and gradually increased to the highest comfortably tolerated level that elicited a clearly visible or palpable pelvic floor muscle contraction, without pain, burning, or unwanted activation of the abdominal, gluteal, or hip adductor muscles. The stimulation intensity achieved during each session was recorded in the treatment documentation [20]. Following electrostimulation, participants completed a biofeedback training session. Using an endovaginal electrode, pelvic floor muscle activity was recorded and displayed in real time as an electromyographic signal. Participants were instructed to perform voluntary contractions and relaxation of the pelvic floor muscles in response to visual cues provided by the training interface. Correct muscle activation resulted in an increase in the on-screen indicator, whereas insufficient or prematurely reduced contraction led to a decrease or absence of signal change. Each contraction lasted 7 s and was followed by a 14-s relaxation period. A single biofeedback training session lasted approximately 5 min [21].

Participants in the VR + ES group received an additional virtual reality session immediately before electrostimulation and biofeedback training. The intervention was delivered using a head-mounted display system (VRTierOne, Stolgraf^®^, Stanowice, Poland). Each session lasted approximately 20 min. The virtual environment was designed as an immersive therapeutic scenario combining visual and auditory stimuli to enhance patient engagement and reduce therapy-related stress. The environment took the form of a garden landscape that gradually evolved over successive sessions. During therapy, participants performed simple tasks such as mandala painting and guided breathing exercises, intended to support relaxation and psychological involvement in the rehabilitation process. All sessions were supervised by a physiotherapist experienced in pelvic floor rehabilitation. The VR intervention was intended as a general adjunct to support relaxation and engagement during rehabilitation rather than as a task-specific pelvic floor training modality linked to pelvic floor muscle activation or real-time EMG feedback. Engagement, satisfaction, acceptability, and perceived presence during the VR sessions were not formally assessed in this study. During electrostimulation, participants were repeatedly asked about discomfort or other adverse sensations throughout the procedure. Following each VR session, participants were asked about their experience and any adverse symptoms.

### 2.7. Outcome Measures

Questionnaire-based outcomes were administered by an assessor blinded to group allocation at three time points: baseline (T0), immediately after completion of the intervention (T1), and at the 2-week follow-up (T2). EMG measurements were performed by the treating physiotherapist using the same equipment employed for biofeedback training and were therefore not collected under blinded conditions. At T0 and T1, EMG was recorded before any electrostimulation or voluntary contraction training during that visit, following a 5-min rest period in the standardized measurement position. At T2, which did not include electrostimulation or training, EMG was recorded after the same 5-min rest period under otherwise identical measurement conditions. Bladder diaries were self-completed by participants and subsequently collected and checked for completeness by the blinded assessor. Given the nature of the VR intervention, participants and the treating physiotherapist could not be blinded to group allocation.

The primary outcome was incontinence-related quality of life, assessed using the Incontinence Quality of Life Questionnaire (I-QOL). The I-QOL is a validated 22-item instrument comprising three domains: avoidance and limiting behavior, psychosocial impact, and social embarrassment. Scores are transformed to a 0–100 scale, with higher scores indicating better quality of life [22,23]. The I-QOL was selected as the primary outcome because it is an incontinence-specific quality-of-life measure with established psychometric properties [22,23].

Secondary outcomes included electromyographic (EMG) parameters of pelvic floor muscle activity, micturition variables, symptom severity, and quality-of-life and psychological outcomes. EMG activity was assessed at rest and during contraction. Pelvic floor muscle activity was assessed using the NeuroTrac^®^ MyoPlus4 Pro system and an intravaginal PR-02 probe. The EMG signal was processed as RMS amplitude (µV) using the device’s band-pass filtering (18–370 Hz or 10–370 Hz, depending on signal amplitude) and a 50-Hz notch filter. Participants performed three 10-s maximal voluntary contractions, each followed by 10 s of rest. Mean Work and Rest values were calculated across the three trials, excluding the first second of each phase [24,25]. The same individually assigned PR-02 intravaginal probe and the same body position were used for each participant at all assessments, and probe insertion depth was standardized using the depth marker on the probe. Micturition data were obtained from bladder diaries completed by participants and included mean, maximum, and minimum micturition volume and micturition frequency [26,27]. The bladder diary was completed over 3 consecutive days at each assessment point. A diary was considered valid if at least 2 of the 3 days were fully completed. For valid diaries with 2 complete days, bladder-diary variables were calculated using the completed days only. Diaries not meeting this criterion would have been excluded from analysis, although no participants were excluded on this basis. Fluid intake was not systematically monitored alongside voiding data. Leakage-related and urgency-related symptoms and pad use were documented separately through the Gaudenz questionnaire and clinical assessment. However, the numbers of leakage and urgency episodes and quantitative pad-use data were not recorded in the bladder diaries and were not analyzed as study outcomes. Accordingly, the analyzed bladder-diary variables do not directly quantify urinary leakage or establish improvement in urinary incontinence itself. Symptom severity and quality of life were evaluated using validated instruments, including the Incontinence Impact Questionnaire Short Form (IIQ-7) [28,29], the King’s Health Questionnaire (KHQ) [30,31], and the Pelvic Organ Prolapse/Urinary Incontinence Sexual Questionnaire (PISQ-12) [32,33]. The PISQ-12 was administered only to participants who reported being sexually active. If sexual activity was not applicable or responses were insufficient to calculate a total score, no total score was derived; the observation was treated as missing and was not imputed.

Psychological outcomes were assessed using the Perceived Stress Scale (PSS-10) and the Rosenberg Self-Esteem Scale (SES) [34,35,36,37]. Validated Polish-language versions of all questionnaire-based outcome measures were used.

### 2.8. Data Analysis

All analyses were performed using JASP v0.96. Baseline characteristics were summarized descriptively as mean (standard deviation, SD) for continuous variables and number (percentage) for categorical variables. Because group allocation was ultimately non-randomized, baseline characteristics were described rather than formally compared inferentially. No missing values were imputed; the mixed-model analyses used all available post-baseline observations under a missing-at-random assumption. Consequently, the number of observations could vary across outcomes and time points. All tests were two-sided, and statistical significance was set at *p* < 0.05. No adjustment for multiple comparisons was planned or applied across the secondary outcomes. Given the number of secondary analyses performed, the corresponding *p* values should be interpreted as exploratory and hypothesis-generating rather than confirmatory.

The primary outcome was the I-QOL total score, and the prespecified primary assessment time point was T1, immediately after the 2-week treatment period. The primary statistical comparison was the baseline-adjusted between-group difference in I-QOL total score at T1 (VR + ES minus ES), estimated using a planned contrast from the mixed model. T2 was an additional 2-week post-treatment follow-up. The group × time interaction was treated as a secondary test of whether the adjusted between-group difference changed between T1 and T2; it did not test change from baseline. Secondary outcomes included the I-QOL subscales, EMG measures, micturition parameters, symptom- and condition-specific quality-of-life outcomes, and psychological outcomes. For each continuous outcome, a separate baseline-adjusted linear mixed model was fitted. Post-baseline observations, that is, post-intervention (T1) and 2-week follow-up (T2), were entered as repeated measures, while the corresponding baseline value (T0) of the same outcome was included as a covariate. In each model, a fixed intercept and fixed effects for group, time, the corresponding baseline score, and the group × time interaction were included, with participant included as a random intercept. Fixed effects were evaluated using Type III tests, and degrees of freedom were estimated using Satterthwaite’s method. Group and time were treated as categorical factors. The omnibus group test and the time-specific planned contrasts address different model hypotheses; the group × time interaction evaluates whether the adjusted between-group difference changes between T1 and T2, while planned contrasts of the estimated marginal means quantify the adjusted between-group difference separately at each post-baseline assessment. All omnibus tests and planned contrasts reported for a given outcome were derived from the same final model.

For the primary outcome, the main inferential analysis was based on the baseline-adjusted linear mixed model described above. Adjusted between-group differences for the post-intervention and 2-week follow-up assessments were obtained from planned contrasts of the estimated marginal means and are presented with 95% confidence intervals (CIs) and two-sided *p* values. As an exploratory sensitivity analysis for the primary outcome, the model was refitted with previous UI-directed intervention (yes/no) included as an additional fixed-effect covariate because previous UI-directed treatment could influence current symptom burden and treatment response and was markedly imbalanced between the groups. Adjusted between-group contrasts at post-intervention and 2-week follow-up were estimated using the same approach as in the primary analysis. Because this was the most clinically relevant baseline imbalance, an additional exploratory sensitivity analysis restricted the primary model to participants without previous UI-directed interventions. Further covariate adjustment was avoided because of the small sample size. The same modeling strategy was applied to secondary continuous outcomes. Baseline values are reported descriptively only.

## 3. Results

### 3.1. Baseline Characteristics of Participants

**Figure 1:**
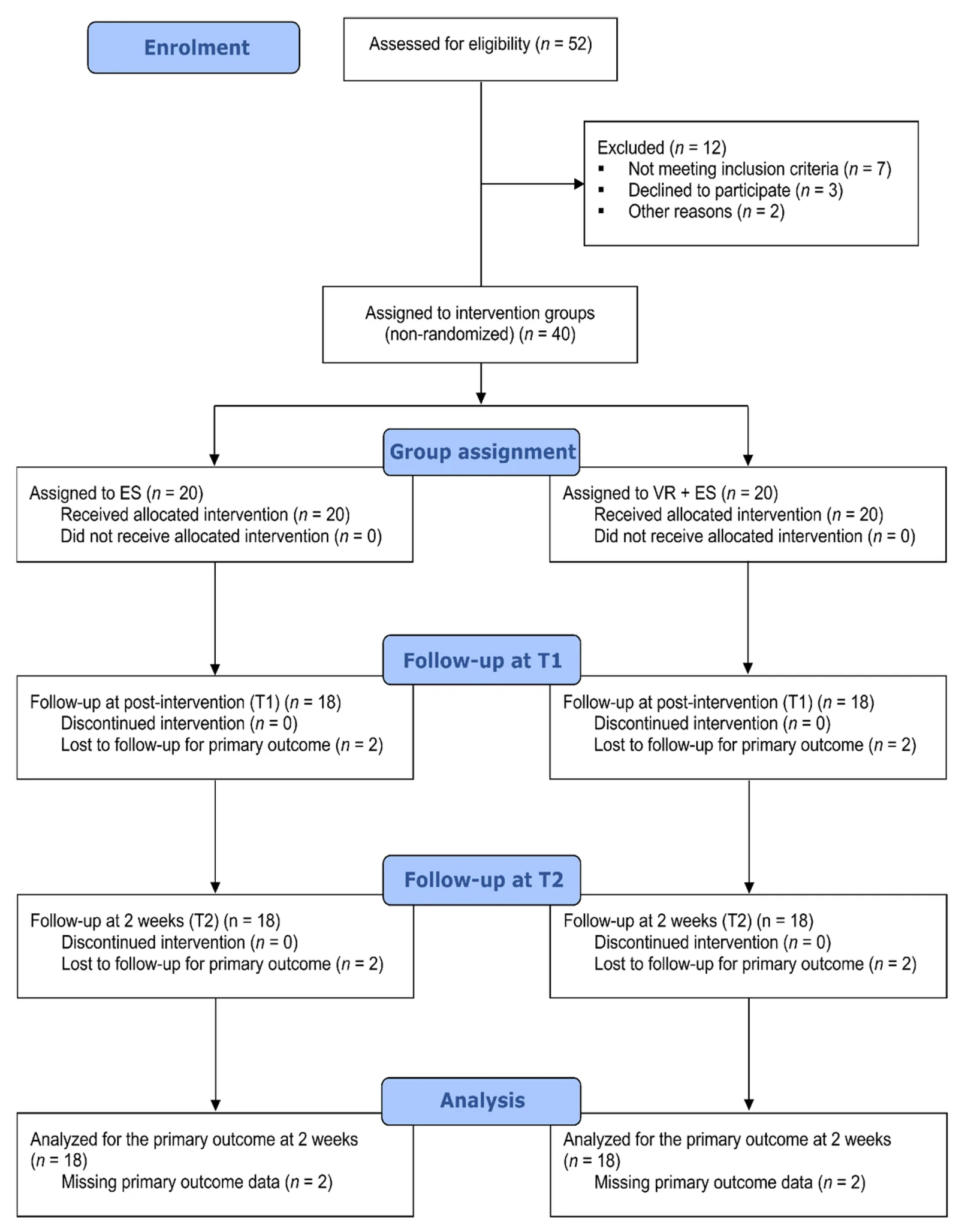
Participant flow diagram.

A total of 52 women were assessed for eligibility, of whom 40 were enrolled and assigned to the ES or VR + ES group, with 20 participants in each group. All participants received the assigned intervention, and no intervention-related adverse events were identified through systematic questioning during the treatment sessions. For the primary outcome, four participants (two per group) were lost to follow-up from the post-intervention assessment onward, resulting in 36 participants (18 per group) with available primary outcome data at both post-intervention and 2-week follow-up. Reasons for loss to follow-up included withdrawal of consent (*n* = 2), relocation or scheduling conflicts (*n* = 1), and unspecified personal reasons (*n* = 1). These reasons were distributed similarly across groups, with one withdrawal in each group and one additional loss in each group due to scheduling or personal reasons. A descriptive review of baseline characteristics showed no clear pattern of differences between participants who completed follow-up and those who were lost to follow-up. Baseline characteristics are presented in Table 1; notable between-group differences were observed for selected clinical characteristics, particularly previous UI-directed intervention history. Eight participants in the VR + ES group had previously undergone a UI-directed intervention: four transobturator tape (TOT) procedures (2019, 2022, 2023, and 2024), three botulinum toxin treatments (January–February 2025), and one laser treatment for UI (February 2025). Exact time intervals in months were not available, and the data did not permit reliable distinction between persistent and recurrent symptoms. Participant flow is shown in Figure 1.

### 3.2. Primary Outcome

Thirty-six participants (18 per group) contributed to the primary I-QOL analysis, providing 72 post-baseline observations. The prespecified primary assessment time point was T1. Baseline I-QOL total score significantly predicted post-baseline outcomes (*p* < 0.01). At T1, the adjusted between-group difference was −1.65 points (95% CI, −10.03 to 6.74; *p* = 0.70); at T2, it was −4.00 points (95% CI, −12.38 to 4.39; *p* = 0.35). No significant main effects of group (*p* = 0.49) or time (*p* = 0.46) were observed, and the group × time interaction, which assesses whether the adjusted between-group difference changed between T1 and T2, was not significant (*p* = 0.41). Detailed observed means and adjusted contrasts are presented in Table 2, and exact omnibus test statistics are reported in Supplementary Table S3. In the exploratory sensitivity analysis additionally adjusted for previous UI-directed intervention, the adjusted between-group difference was −0.14 points (95% CI, −9.91 to 9.64; *p* = 0.98) at post-intervention and −2.49 points (95% CI, −12.26 to 7.29; *p* = 0.62) at the 2-week follow-up. The group × time interaction remained non-significant (*p* = 0.40). A second exploratory sensitivity analysis restricted to participants without previous UI-directed interventions (*n* = 28; ES, *n* = 18; VR + ES, *n* = 10) yielded an adjusted between-group difference of −1.89 points (95% CI, −12.27 to 8.49; *p* = 0.72) at T1 and −0.70 points (95% CI, −11.07 to 9.68; *p* = 0.90) at T2. Given the reduced sample size and non-randomized design, this analysis remains exploratory.

A similar pattern was found across the three I-QOL subscales. Baseline subscale scores significantly predicted post-baseline outcomes in all models (all *p* < 0.01), whereas no significant group × time interaction was detected for ALB, PSI, or SE (*p* = 0.19, *p* = 1.00, and *p* = 0.31, respectively). No significant main effect of group was observed in any of the subscale models. Adjusted between-group contrasts for all three subscales at post-intervention and 2-week follow-up are reported in Table 2.

### 3.3. Secondary Outcomes

Complete omnibus fixed-effect tests for all secondary outcomes are reported in Supplementary Table S3; adjusted between-group contrasts and their 95% CIs are reported in Supplementary Table S2.

#### 3.3.1. EMG Outcomes

**Figure 2:**
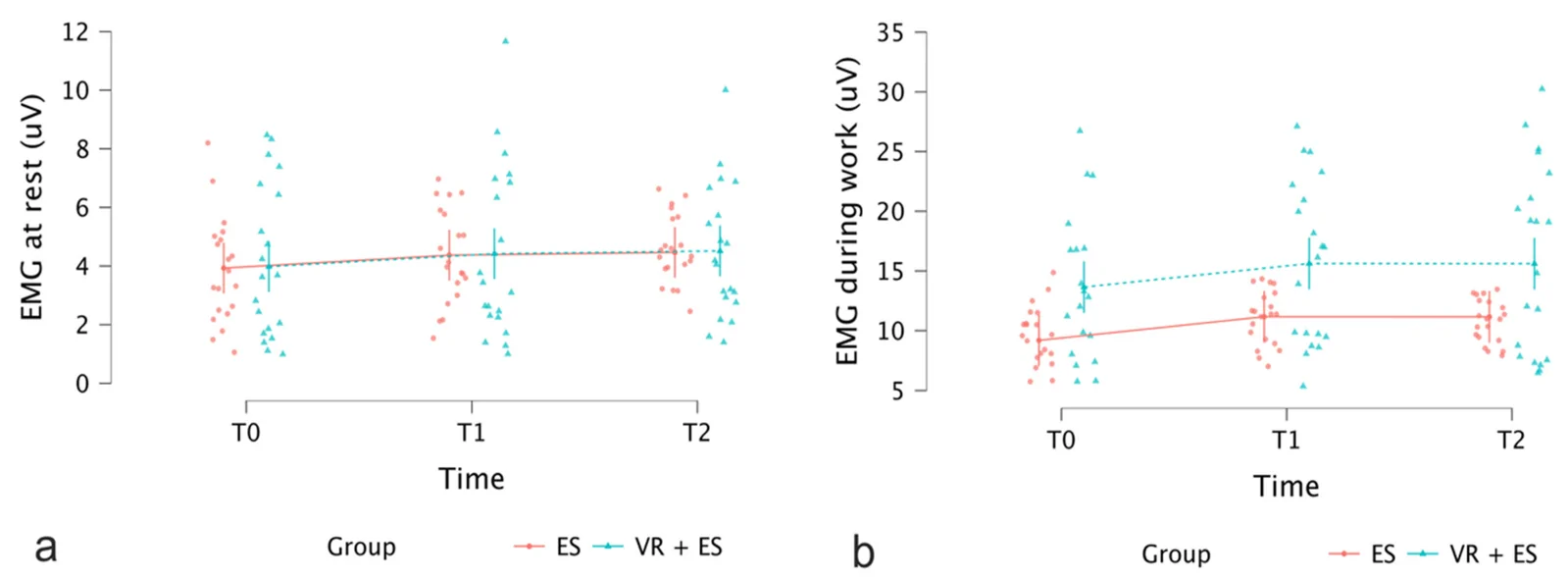
Observed EMG outcomes across study time points. Panel (**a**) shows EMG at rest, and panel (**b**) shows EMG during work. Points represent individual observations. Lines represent observed group means, and error bars indicate 95% confidence intervals for the observed means.

For EMG at rest, baseline EMG significantly predicted post-baseline values (*p* < 0.01). No significant main effect of group was observed (*p* = 0.70), and neither the effect of time nor the group × time interaction reached significance. The same pattern was found for EMG during work: baseline values significantly predicted follow-up scores (*p* < 0.01), the main effect of group did not reach statistical significance (*p* = 0.051), while no significant effect of time or group × time interaction was detected. In both EMG models, adjusted between-group contrasts at post-intervention and 2-week follow-up were non-significant. Observed values over time are shown in Figure 2, and detailed adjusted contrasts are presented in Supplementary Table S2.

#### 3.3.2. Symptom and Quality-of-Life Outcomes

**Figure 3:**
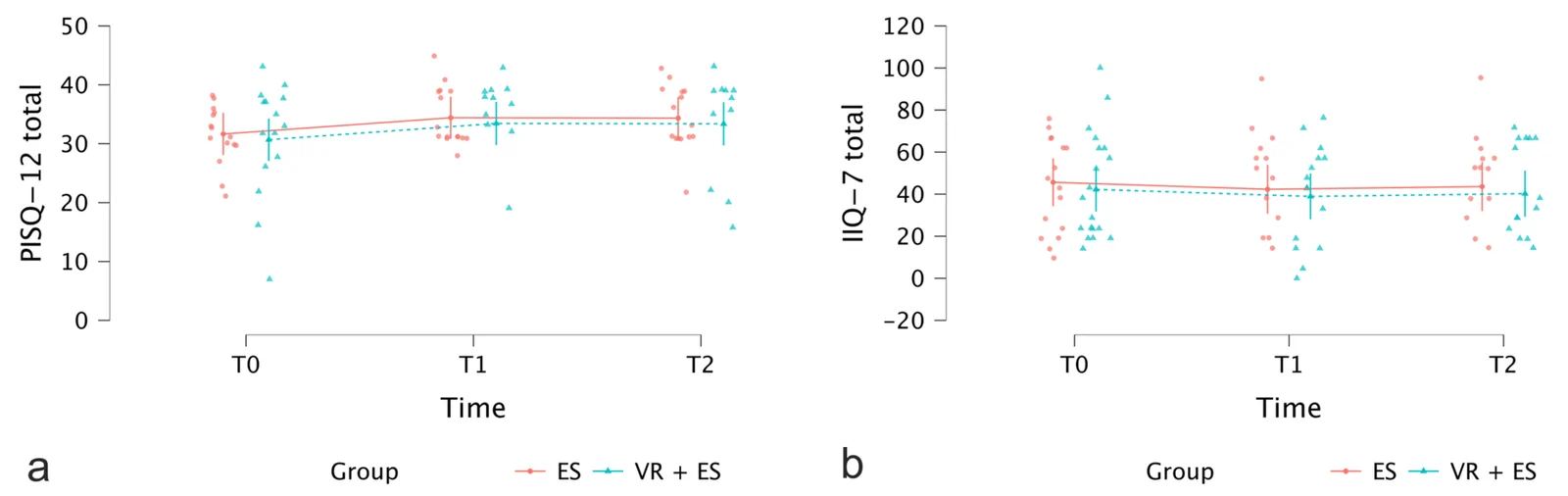
Observed symptom and condition-specific quality-of-life outcomes across study time points. Panel (**a**) shows PISQ-12 Total, and panel (**b**) shows IIQ-7 Total. Points represent individual observations. Lines represent observed group means, and error bars indicate 95% confidence intervals for the observed means.

For IIQ-7 Total and PISQ-12 Total, baseline scores were significant predictors of post-baseline outcomes in both models (both *p* < 0.01). IIQ-7 Total showed no significant main effects of group or time, and no significant group × time interaction (*p* = 0.73); adjusted between-group contrasts at post-intervention and 2 weeks were likewise non-significant. In the PISQ-12 model, no significant main effect of group was identified (*p* = 0.53); neither time nor the interaction term reached significance, and the adjusted between-group contrasts remained non-significant at both follow-up assessments. Across KHQ domains, no consistent pattern of significant group × time effects was observed. Observed values for IIQ-7 Total and PISQ-12 Total are shown in Figure 3, and detailed adjusted contrasts for IIQ-7, PISQ-12, and KHQ domains are presented in Supplementary Table S2.

#### 3.3.3. Micturition Outcomes

**Figure 4:**
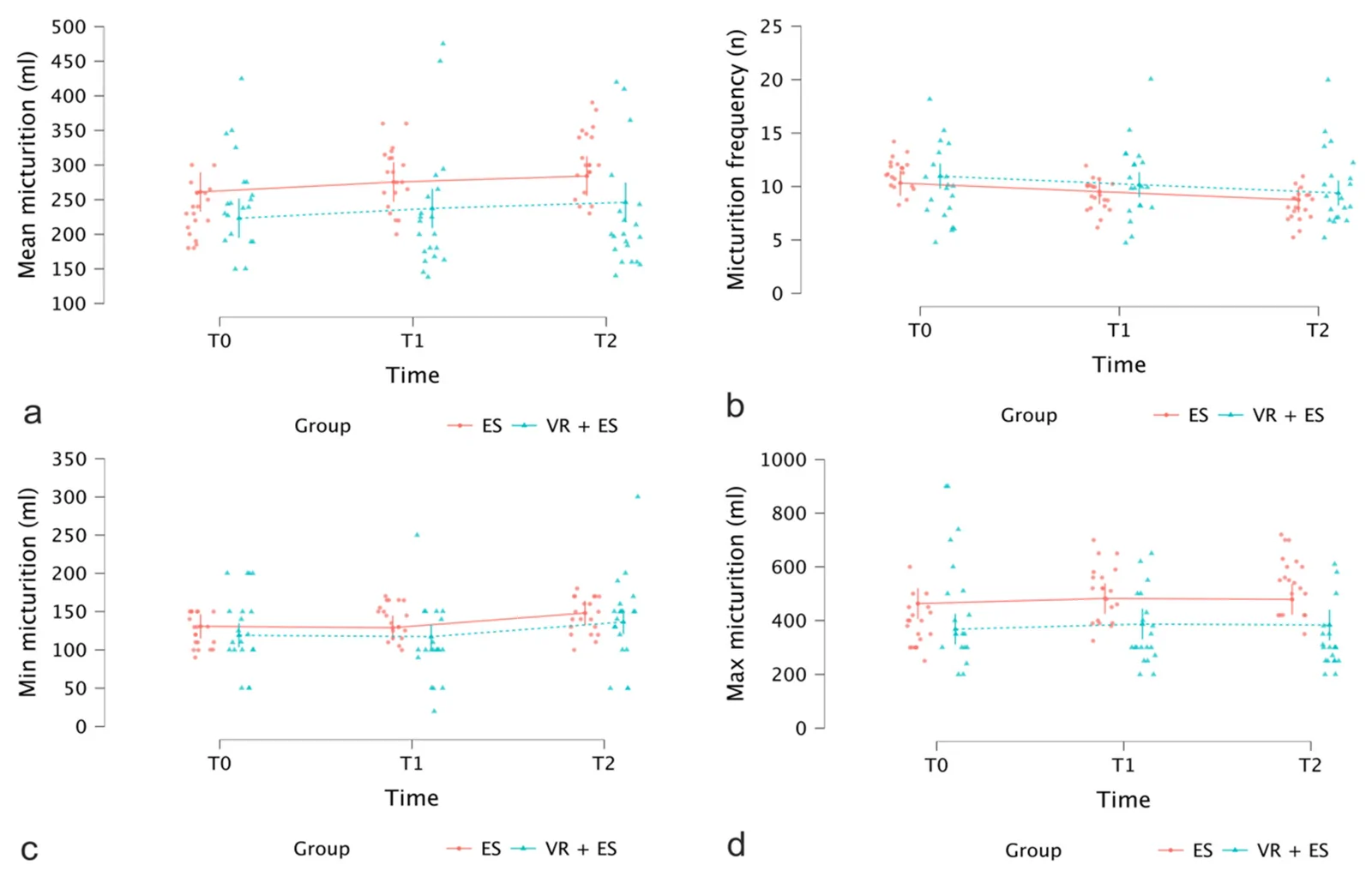
Observed micturition outcomes across study time points. Panel (**a**) shows mean micturition volume (mL), panel (**b**) shows micturition frequency, panel (**c**) shows minimum micturition volume (mL), and panel (**d**) shows maximum micturition volume (mL). Points represent individual observations. Lines represent observed group means, and error bars indicate 95% confidence intervals for the observed means.

For micturition-related outcomes, baseline values significantly predicted post-baseline scores for mean micturition volume, maximum micturition volume, and micturition frequency (all *p* < 0.01), but not for minimum micturition volume (*p* = 0.14). Mean micturition volume showed a significant main effect of group (*p* < 0.001), while the group × time interaction did not reach significance (*p* = 0.07); adjusted between-group contrasts were nevertheless significant at both follow-up assessments, with lower values in the VR + ES group than in the ES group. For maximum micturition volume, both the main effect of group and the group × time interaction were significant (group effect, *p* < 0.001; group × time interaction, *p* = 0.008), and adjusted between-group contrasts were significant at post-intervention and at 2 weeks, again with lower values in the VR + ES group. For minimum micturition volume, the group × time interaction was significant (*p* = 0.048). The adjusted between-group contrast was significant at post-intervention, with a lower value in the VR + ES group, but was not significant at the 2-week follow-up. No significant main effect of group was observed (*p* = 0.12), whereas the main effect of time remained significant (*p* = 0.003). Micturition frequency showed significant main effects of group (*p* < 0.001) and time (*p* = 0.005) without a significant interaction (*p* = 0.39), while adjusted between-group contrasts were significant at both follow-up assessments, with higher frequency in the VR + ES group than in the ES group. Observed values over time are shown in Figure 4, and detailed adjusted contrasts are presented in Supplementary Table S2. These secondary comparisons were exploratory and were not adjusted for multiple comparisons. Because the analyzed bladder-diary variables comprised voided volume and micturition frequency rather than quantitative leakage episodes, they cannot be interpreted as direct evidence of reduced urinary leakage or improvement in urinary incontinence itself.

#### 3.3.4. Psychological Outcomes

**Figure 5:**
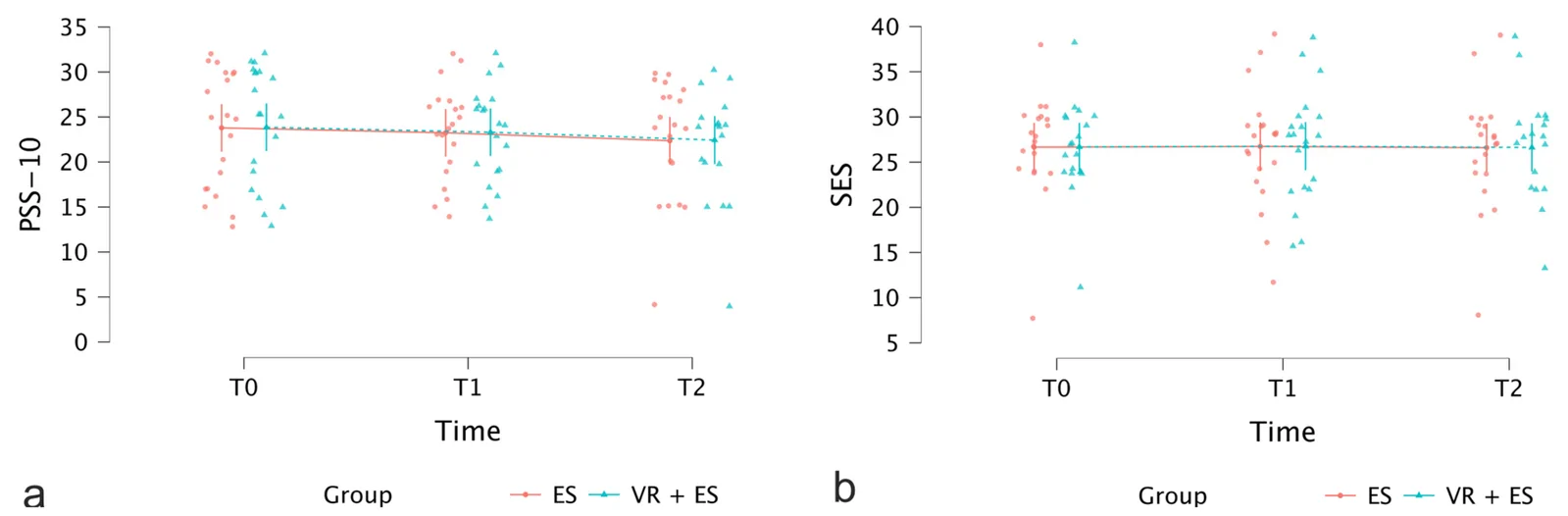
Observed psychological outcomes across study time points. Panel (**a**) shows PSS-10, and panel (**b**) shows SES. Points represent individual observations. Lines represent observed group means, and error bars indicate 95% confidence intervals for the observed means.

For both PSS-10 and SES, baseline scores significantly predicted post-baseline outcomes (both *p* < 0.01). In the PSS-10 model, no significant main effect of group was observed (*p* = 0.60); neither time nor the group × time interaction was significant, and adjusted between-group contrasts at both follow-up assessments were non-significant. For SES, neither the main effects nor the interaction term reached significance, and no significant adjusted between-group contrasts were observed at post-intervention or at 2 weeks. Observed values over time are shown in Figure 5, and detailed adjusted contrasts are presented in Supplementary Table S2.

## 4. Discussion

The aim of this study was to assess whether adding virtual reality to pelvic floor muscle electrostimulation with EMG biofeedback was associated with additional clinical benefits in women with urinary incontinence. In this non-randomized controlled study, we did not detect a statistically significant additional improvement in the primary outcome, that is, incontinence-related quality of life measured with the I-QOL total score. The exploratory sensitivity analysis additionally adjusting for previous UI-directed intervention yielded similar between-group estimates and did not materially change the primary finding. The additional sensitivity analysis restricted to participants without previous UI-directed interventions was also consistent with the primary analysis. Similarly, no significant effects were observed across the I-QOL subscales. The absence of a statistically significant between-group difference in the primary outcome may be related to several factors. First, both groups received active pelvic floor rehabilitation consisting of electrostimulation and EMG biofeedback. Previous studies have demonstrated that pelvic floor muscle training, particularly when supported by biofeedback, can lead to improvements in urinary symptoms and quality of life in women with urinary incontinence [38,39]. While electrostimulation combined with EMG biofeedback directly targets pelvic floor muscle activation and neuromuscular control, the VR component was primarily designed to enhance patient engagement and provide a relaxing, immersive environment rather than to deliver task-specific pelvic floor training. The VR component was based primarily on relaxation- and therapeutic-garden approaches previously investigated for psychological outcomes [40,41]. Unlike electrostimulation and EMG biofeedback, it did not provide task-specific pelvic floor training. This may have limited its potential to influence pelvic floor-specific outcomes.

Among secondary outcomes, no significant adjusted between-group contrasts at either follow-up assessment were observed for EMG measures, IIQ-7 Total, PISQ-12 Total, perceived stress, or self-esteem. Consistently, no significant main effects of group or group × time interactions were observed for these outcomes. In contrast, several micturition-related outcomes differed between groups. Specifically, the VR + ES group showed lower mean and maximum micturition volumes and higher micturition frequency than the ES group at both follow-up assessments, whereas the difference in minimum micturition volume was limited to the post-intervention assessment. These findings should be interpreted cautiously, as bladder-diary parameters can exhibit substantial intra-individual variability and may be influenced by behavioral and environmental factors [42,43]. In addition, the analyzed diary variables did not directly quantify urinary leakage, and fluid intake was not systematically recorded.

With respect to clinical significance, previously reported MCID values for the I-QOL suggest a between-treatment difference of approximately 2.5 points and a within-treatment change of approximately 6.3 points [44]. These thresholds were derived primarily from women with stress urinary incontinence, and their applicability to the clinically heterogeneous population included in the present study is uncertain. In the present study, the adjusted between-group difference at 2-week follow-up was −4.00 points (95% CI −12.38 to 4.39). The wide confidence interval included both negligible and potentially clinically meaningful effects in either direction, indicating substantial uncertainty in the effect estimate and precluding a clear conclusion regarding the presence or absence of an additional effect of VR.

Overall, the findings do not support a clear additional benefit of virtual reality for the primary quality-of-life outcome in this sample. However, although differences were observed in selected micturition parameters, their direction did not indicate a consistent advantage of VR + ES over ES. These findings should therefore be interpreted cautiously, particularly given the non-randomized design and baseline between-group differences in selected clinical characteristics. This distinction may be particularly relevant in rehabilitation, where task-specific practice and appropriate feedback are important components of VR-based motor training [45], while specific pelvic floor muscle training remains the exercise approach with the strongest evidence for SUI [46].

In addition, the relatively short duration of the intervention and the limited number of sessions may have reduced the likelihood of detecting potential additive effects of VR. Moreover, because the VR component focused primarily on relaxation and engagement rather than task-specific pelvic floor training, patient experience, engagement, and acceptability would have been relevant outcomes but were not formally assessed in this study.

### Limitations

This study has several limitations. Most importantly, although the study was initially planned and submitted for registration as a randomized controlled trial, randomization could not be implemented, and allocation was ultimately non-randomized and unconcealed. This protocol deviation increases the risk of selection bias and residual confounding and limits causal interpretation of the between-group estimates. Because the groups were recruited sequentially rather than concurrently, confounding related to calendar time or other unmeasured time-dependent circumstances cannot be excluded. This concern is particularly relevant given the baseline differences between groups in previous UI-directed intervention, overall surgical history, BMI, and baseline I-QOL scores. Although outcome-specific baseline adjustment was applied and the primary-outcome sensitivity analyses accounted for previous UI-directed intervention history, including an analysis restricted to participants without such interventions, these approaches cannot fully account for confounding arising from other measured or unmeasured baseline differences. The inclusion of women with SUI, UUI, and MUI also introduced clinical heterogeneity, and the small numbers within individual UI subtypes precluded meaningful subtype-specific or adjusted analyses. The same standardized stimulation protocol was applied across UI subtypes. However, evidence supporting these specific stimulation parameters is strongest for SUI, which further limits generalization to UUI and heterogeneous MUI populations.

Several aspects of outcome measurement should also be considered. Participants and the treating physiotherapist could not be blinded to group allocation because of the nature of the intervention, although questionnaire-based outcomes were administered by an assessor blinded to group allocation. EMG measurements were performed by the treating physiotherapist and were therefore not collected under blinded conditions. Bladder-diary outcomes were participant-recorded and may be affected by day-to-day behavioral variability; moreover, fluid intake was not systematically monitored, which limits interpretation of the observed differences in voiding volume and frequency. The bladder-diary variables therefore cannot be interpreted as direct measures of reduced urinary leakage. Missing outcome data were analyzed without imputation under a missing-at-random assumption; because this assumption cannot be verified, attrition-related bias cannot be excluded.

Finally, the study included a small exploratory sample and no formal a priori power calculation was performed; consequently, the study was not designed to estimate clinically meaningful between-group differences with sufficient precision. In addition, numerous secondary outcomes were examined without adjustment for multiple comparisons, and statistically significant secondary findings should therefore be regarded as exploratory. The intervention period and follow-up were short, limiting assessment of longer-term effects. Participants in the VR + ES group also received approximately 20 additional minutes of supervised intervention per session, meaning that the design cannot fully distinguish the specific effect of immersive VR from non-specific effects of additional therapeutic exposure and attention. Furthermore, VR was delivered before, rather than integrated with, electrostimulation and EMG biofeedback and consisted primarily of relaxation and engagement tasks rather than task-specific pelvic floor training. Taken together, these limitations indicate that the present findings should be considered preliminary and hypothesis-generating rather than definitive evidence regarding the efficacy of adjunctive VR.

## 5. Conclusions

In this small non-randomized pilot study, we did not detect a statistically significant additional improvement in incontinence-related quality of life with adjunctive relaxation-oriented immersive VR delivered before electrostimulation and EMG biofeedback. The confidence intervals around the primary estimates were wide and did not exclude potentially meaningful effects in either direction. Differences observed in selected voiding parameters did not consistently favor the VR-assisted intervention and should be interpreted cautiously. Given the exploratory design, small sample, and potential for residual confounding, the findings should be considered preliminary and hypothesis-generating. Task-specific VR integrated with real-time pelvic floor EMG feedback was not evaluated in the present study and should be considered a separate hypothesis for future randomized research with longer follow-up.

## Figures and Tables

**Table 1:** Baseline characteristics of participants.

Variable		Total	ES	VR + ES
*n*		40	20	20
Age, years		63.0 (12.7)	62.1 (14.7)	63.9 (10.8)
Body mass, kg		72.3 (14.1)	69.1 (13.1)	75.5 (14.8)
Body height, cm		161.4 (6.1)	161.8 (4.5)	161.1 (7.4)
BMI, kg/m^2^		27.8 (5.7)	26.5 (5.5)	29.2 (5.7)
Parity, *n* of births		2.0 (0.7)	2.2 (0.7)	1.8 (0.7)
Incontinence duration, years		16.3 (14.1)	16.1 (13.0)	16.5 (15.3)
Any prior surgery, yes, *n* (%)		34 (85.0)	20 (100.0)	14 (70.0)
Previous UI-directed intervention, yes, *n* (%)		8 (20.0)	0 (0.0)	8 (40.0)
Mode of delivery, *n* (%)				
	Vaginal delivery only	27 (67.5)	15 (75.0)	12 (60.0)
	Assisted vaginal delivery	2 (5.0)	2 (10.0)	0 (0.0)
	Cesarean delivery only	4 (10.0)	2 (10.0)	2 (10.0)
	Both vaginal and cesarean delivery	6 (15.0)	1 (5.0)	5 (25.0)
	Not reported	1 (2.5)	0 (0.0)	1 (5.0)
Urinary incontinence subtype, *n* (%)				
	Stress urinary incontinence	7 (17.5)	5 (25.0)	2 (10.0)
	Urgency urinary incontinence	4 (10.0)	1 (5.0)	3 (15.0)
	Mixed urinary incontinence	29 (72.5)	14 (70.0)	15 (75.0)
I-QOL total score at T0 (0–100)		45.4 (28.1)	49.3 (30.1)	41.2 (26.0)

Data are presented as mean (SD) or *n* (%). Percentages are column percentages. For incontinence duration, data were available for 38 participants (ES, *n* = 18; VR + ES, *n* = 20). ES: electrostimulation; VR: virtual reality; VR + ES: virtual reality plus electrostimulation; BMI: body mass index; UI: urinary incontinence; I-QOL: Incontinence Quality of Life Questionnaire.

**Table 2:** Observed I-QOL scores and baseline-adjusted between-group differences at post-intervention and 2-week follow-up.

Variable	Time Point	ES, Mean ± SD	VR + ES, Mean ± SD	Adjusted Mean Difference, VR + ES vs. ES (95% CI)	*p* Value
I-QOL total	Baseline	49.3 ± 30.1	41.2 ± 26.0	–	–
	Post	53.8 ± 26.4	45.6 ± 26.0	−1.65 (−10.03 to 6.74)	0.70
	2 weeks	56.0 ± 23.5	45.5 ± 28.6	−4.00 (−12.38 to 4.39)	0.35
I-QOL ALB	Baseline	49.2 ± 30.2	43.0 ± 25.	–	–
	Post	58.1 ± 27.9	49.1 ± 25.9	−4.09 (−13.69 to 5.52)	0.40
	2 weeks	62.1 ± 24.6	48.6 ± 28.4	−8.63 (−18.24 to 0.97)	0.08
I-QOL PSI	Baseline	52.0 ± 31.1	41.7 ± 27.0	–	–
	Post	52.2 ± 26.3	44.3 ± 26.8	0.78 (−7.47 to 9.03)	0.85
	2 weeks	53.9 ± 23.6	46.0 ± 29.8	0.78 (−7.47 to 9.03)	0.85
I-QOL SE	Baseline	44.7 ± 31.2	37.5 ± 28.2	–	–
	Post	49.7 ± 29.9	42.2 ± 27.8	−2.68 (−14.92 to 9.56)	0.67
	2 weeks	50.0 ± 28.9	39.4 ± 30.3	−5.74 (−17.98 to 6.50)	0.36

Data are presented as observed mean ± SD. Available sample sizes for the I-QOL outcomes were ES/VR + ES: 19/18 at baseline, 18/18 at post-intervention, and 18/18 at the 2-week follow-up. For each I-QOL model, 36 participants (18 per group) and 72 post-baseline observations contributed to the mixed-model analysis. T1 was the prespecified primary assessment time point; T2 was an additional follow-up. Adjusted mean differences at post-intervention and 2-week follow-up were estimated using baseline-adjusted linear mixed models with a fixed intercept, fixed effects for group, time, baseline score, and the group × time interaction, and a random intercept for participant. Positive values indicate higher scores in the VR + ES group relative to the ES group. ES, electrostimulation; VR + ES, virtual reality plus electrostimulation; I-QOL, Incontinence Quality of Life Questionnaire; ALB, avoidance and limiting behavior; PSI, psychosocial impact; SE, social embarrassment.

## Data Availability

The data are not publicly available because the dataset contains detailed clinical and demographic information from a small sample, which may pose a risk of participant re-identification. De-identified data are available from the corresponding author upon reasonable request and subject to appropriate safeguards for participant confidentiality.

## Supplementary Materials

The following supporting information can be downloaded at https://www.mdpi.com/article/10.3390/jcm15197726/s1, Supplementary Table S1. Comparison of the original registration with the study as conducted; Table S2: Observed secondary outcomes at baseline, post-intervention, and 2-week follow-up, with baseline-adjusted between-group contrasts; Table S3: Type III fixed-effect tests from the baseline-adjusted linear mixed models.

## Abbreviations

The following abbreviations are used in this manuscript:

ALB	Avoidance and limiting behavior
BMI	Body mass index
CI	Confidence interval
EMG	Electromyography
ES	Electrostimulation with EMG biofeedback
ICS	International Continence Society
IIQ-7	Incontinence Impact Questionnaire—Short Form
I-QOL	Incontinence Quality of Life Questionnaire
KHQ	King’s Health Questionnaire
PISQ-12	Pelvic Organ Prolapse/Urinary Incontinence Sexual Questionnaire—Short Form
PSI	Psychosocial impact
PSS-10	Perceived Stress Scale—10-item version
SD	Standard deviation
SE	Social embarrassment
SES	Rosenberg Self-Esteem Scale
T0	Baseline assessment
T1	Post-intervention assessment
T2	Two-week follow-up assessment
TREND	Transparent Reporting of Evaluations with Nonrandomized Designs
UI	Urinary incontinence
VR	Virtual reality
VR + ES	Virtual reality combined with electrostimulation and EMG biofeedback
