# Supplementary material for: Adjunctive Immersive Virtual Reality for Urinary Incontinence-Related Quality of Life in Postmenopausal Women with Urinary Incontinence: A Non-Randomized Controlled Pilot Trial
Source: J Clin Med. 2026 Oct 7;15(19):7726. doi: 10.3390/jcm15197726 (PMC13666500; doi:10.3390/jcm15197726)
Supplement: Supplementary file 1 [file jcm-15-07726-s001.zip › jcm-4487378-supplementary.pdf]

## Supplementary materials

**Supplementary Table S1.** Comparison of the original registration with the study as conducted.

| Domain | Original registration | Study as conducted | Explanation |
|---|---|---|---|
| **Allocation and enrollment** | Randomized parallel-group allocation; target enrollment of 40 participants. | Non-randomized sequential allocation according to VR-system availability; 40 participants were enrolled, with 20 in each group. | The allocation procedure was changed before enrollment because the VR system was available only for a limited loan period. No outcome data were available at that time. |
| **Interventions** | Both groups received electrostimulation with EMG biofeedback; the experimental group additionally received VR. The original description incorrectly suggested integration of VR with EMG biofeedback. | VR was delivered as a separate session immediately before electrostimulation and EMG biofeedback. The VR system was not technically integrated with the EMG device. | The intervention procedures were not changed during the study. The post-study registry update corrected the original description. |
| **Outcomes and assessments** | The I-QOL score at the end of the 2-week intervention was registered as the primary outcome. Secondary outcomes were specified only for the end-of-treatment assessment. | The I-QOL total score at T1 remained the primary outcome and primary assessment. An additional T2 follow-up and a revised set of secondary outcomes were reported. | The primary outcome and primary assessment were unchanged. The registry was updated after study completion to reflect the secondary outcomes and assessments included in the manuscript. Outcome data had already been examined; these analyses were treated as exploratory. |
| **Statistical analysis** | No detailed statistical analysis plan was included in the registration. | Statistical analyses were conducted as described in Section 2.8. Additional sensitivity analyses requested during peer review were identified as exploratory. | No prospectively registered analysis plan was amended, and the detailed model specification is not presented as prospectively specified. |

**Supplementary Table S2.** Observed secondary outcomes at baseline, post-intervention, and 2-week follow-up, with baseline-adjusted between-group contrasts.

| Variable | Time point | ES, *n* | ES, mean ± SD | VR + ES, n | VR + ES, mean ± SD | Adjusted mean difference, VR + ES vs ES (95% CI) | *P* value |
|---|---|---|---|---|---|---|---|
| EMG rest (μV) | Baseline | 20 | 3.8 ± 1.8 | 20 | 4.1 ± 2.5 | – | – |
| | Post | 20 | 4.4 ± 1.7 | 20 | 4.4 ± 2.9 | -0.08 (-1.23 to 1.07) | 0.888 |
| | 2 weeks | 20 | 4.6 ± 1.2 | 20 | 4.4 ± 2.2 | -0.27 (-1.42 to 0.88) | 0.642 |
| EMG work (μV) | Baseline | 20 | 9.6 ± 2.4 | 20 | 13.2 ± 6.4 | – | – |
| | Post | 20 | 11.0 ± 2.2 | 20 | 15.7 ± 6.7 | 2.25 (-0.53 to 5.03) | 0.113 |
| | 2 weeks | 20 | 10.9 ± 1.8 | 20 | 15.9 ± 7.8 | 2.63 (-0.15 to 5.41) | 0.064 |
| Mean micturition volume (mL) | Baseline | 20 | 234.8 ± 37.0 | 20 | 249.7 ± 69.3 | – | – |
| | Post | 20 | 281.2 ± 45.2 | 20 | 231.6 ± 90.6 | -63.42 (-92.74 to -34.11) | <0.001 |
| | 2 weeks | 20 | 304.8 ± 46.7 | 20 | 225.5 ± 82.3 | -93.02 (-122.34 to -63.71) | <0.001 |
| Maximum micturition volume (mL) | Baseline | 20 | 385.5 ± 87.3 | 20 | 445.5 ± 215.5 | – | – |
| | Post | 20 | 501.2 ± 103.6 | 20 | 367.5 ± 133.4 | -151.76 (-217.81 to -85.70) | <0.001 |
| | 2 weeks | 20 | 537.0 ± 107.0 | 20 | 325.0 ± 114.0 | -230.01 (-296.06 to -163.95) | <0.001 |
| Minimum micturition volume (mL) | Baseline | 20 | 124.5 ± 21.1 | 20 | 125.2 ± 48.7 | – | – |
| | Post | 20 | 138.2 ± 22.3 | 20 | 108.0 ± 49.7 | -30.42 (-55.60 to -5.24) | 0.018 |
| | 2 weeks | 20 | 145.0 ± 23.5 | 20 | 139.5 ± 57.0 | -5.67 (-30.85 to 19.51) | 0.659 |
| Micturition frequency | Baseline | 20 | 11.2 ± 1.4 | 20 | 10.1 ± 3.5 | – | – |
| | Post | 20 | 9.2 ± 1.4 | 20 | 10.5 ± 3.5 | 2.12 (0.92 to 3.31) | <0.001 |
| | 2 weeks | 20 | 8.2 ± 1.4 | 20 | 9.9 ± 3.6 | 2.57 (1.37 to 3.76) | <0.001 |
| KHQ General health perceptions | Baseline | 18 | 54.2 ± 23.1 | 18 | 47.2 ± 19.0 | – | – |
| | Post | 15 | 51.7 ± 22.1 | 16 | 42.2 ± 15.1 | -4.56 (-17.12 to 7.99) | 0.476 |
| | 2 weeks | 15 | 46.7 ± 24.8 | 15 | 40.0 ± 18.4 | -4.60 (-17.27 to 8.08) | 0.477 |
| KHQ Incontinence impact | Baseline | 18 | 68.5 ± 33.3 | 18 | 66.7 ± 22.9 | – | – |
| | Post | 15 | 60.0 ± 31.4 | 16 | 64.6 ± 25.7 | 5.91 (-13.28 to 25.10) | 0.546 |

| | | | | | | | |
|---|---|---|---|---|---|---|---|
| | 2 weeks | 15 | 60.0 ± 31.4 | 14 | 66.7 ± 18.5 | 6.04 (-13.27 to 25.34) | 0.54 |
| KHQ Role limitations | Baseline | 18 | 55.6 ± 33.3 | 17 | 49.0 ± 19.1 | – | – |
| | Post | 15 | 50.0 ± 31.5 | 15 | 43.3 ± 25.8 | -2.83 (-22.66 to 16.99) | 0.779 |
| | 2 weeks | 15 | 48.9 ± 32.4 | 15 | 48.9 ± 18.4 | 2.82 (-17.10 to 22.73) | 0.782 |
| KHQ Physical limitations | Baseline | 18 | 62.0 ± 32.7 | 17 | 63.7 ± 30.2 | – | – |
| | Post | 15 | 60.0 ± 31.4 | 16 | 56.3 ± 29.1 | 0.59 (-20.09 to 21.27) | 0.955 |
| | 2 weeks | 15 | 58.9 ± 30.1 | 15 | 54.5 ± 23.1 | -2.00 (-22.80 to 18.79) | 0.85 |
| KHQ Social limitations | Baseline | 18 | 46.9 ± 31.6 | 17 | 36.6 ± 28.0 | – | – |
| | Post | 15 | 41.5 ± 32.9 | 16 | 29.9 ± 24.3 | 5.36 (-11.49 to 22.21) | 0.533 |
| | 2 weeks | 15 | 45.9 ± 28.1 | 15 | 28.1 ± 27.2 | -0.75 (-17.72 to 16.22) | 0.931 |
| KHQ Personal relationships | Baseline | 18 | 46.3 ± 37.7 | 16 | 34.4 ± 34.1 | – | – |
| | Post | 15 | 37.8 ± 40.1 | 11 | 28.8 ± 39.5 | -5.03 (-31.05 to 20.99) | 0.705 |
| | 2 weeks | 15 | 41.1 ± 36.1 | 12 | 30.6 ± 38.2 | -3.54 (-29.54 to 22.46) | 0.79 |
| KHQ Emotions | Baseline | 18 | 59.9 ± 35.6 | 17 | 54.9 ± 28.5 | – | – |
| | Post | 15 | 54.8 ± 33.7 | 16 | 54.2 ± 31.7 | 1.21 (-18.59 to 21.01) | 0.905 |
| | 2 weeks | 15 | 55.6 ± 35.6 | 15 | 52.6 ± 24.7 | -1.92 (-21.87 to 18.04) | 0.851 |
| KHQ Sleep/Energy | Baseline | 18 | 50.9 ± 31.0 | 18 | 62.0 ± 26.7 | – | – |
| | Post | 15 | 48.9 ± 27.8 | 16 | 49.0 ± 30.7 | -5.78 (-21.87 to 10.30) | 0.481 |
| | 2 weeks | 15 | 46.7 ± 28.3 | 15 | 47.8 ± 31.4 | -5.71 (-21.89 to 10.47) | 0.489 |
| KHQ Severity measures | Baseline | 18 | 63.9 ± 26.7 | 18 | 70.8 ± 23.4 | – | – |
| | Post | 15 | 66.1 ± 23.7 | 15 | 62.2 ± 24.2 | -4.81 (-21.39 to 11.78) | 0.57 |
| | 2 weeks | 15 | 66.1 ± 24.3 | 11 | 59.8 ± 25.5 | -4.16 (-20.89 to 12.57) | 0.626 |
| IIQ-7 Physical activity | Baseline | 16 | 55.2 ± 30.9 | 19 | 36.0 ± 28.5 | – | – |
| | Post | 13 | 52.6 ± 26.2 | 15 | 36.7 ± 26.9 | -4.15 (-18.91 to 10.62) | 0.582 |
| | 2 weeks | 13 | 53.8 ± 21.7 | 14 | 36.9 ± 25.5 | -5.41 (-20.21 to 9.39) | 0.474 |
| IIQ-7 Travel | Baseline | 16 | 31.2 ± 34.4 | 19 | 36.8 ± 35.8 | – | – |

| | | | | | | | |
|---|---|---|---|---|---|---|---|
| | Post | 13 | 47.4 ± 37.2 | 15 | 28.9 ± 24.8 | -20.35 (-42.67 to 1.98) | 0.074 |
| | 2 weeks | 13 | 46.2 ± 33.4 | 14 | 41.7 ± 33.2 | -8.05 (-30.60 to 14.51) | 0.484 |
| IIQ-7 Social relationships | Baseline | 16 | 27.1 ± 34.9 | 19 | 40.3 ± 37.8 | – | – |
| | Post | 13 | 18.0 ± 35.0 | 15 | 35.5 ± 34.4 | 9.78 (-13.00 to 32.56) | 0.4 |
| | 2 weeks | 13 | 18.0 ± 35.0 | 14 | 30.9 ± 30.6 | 4.87 (-18.21 to 27.94) | 0.679 |
| IIQ-7 Emotional health | Baseline | 16 | 53.1 ± 34.5 | 19 | 60.5 ± 33.0 | – | – |
| | Post | 13 | 60.3 ± 29.3 | 15 | 55.6 ± 39.2 | -5.32 (-17.36 to 6.73) | 0.387 |
| | 2 weeks | 13 | 61.5 ± 29.2 | 14 | 57.1 ± 35.0 | -7.86 (-20.10 to 4.38) | 0.208 |
| IIQ-7 Total | Baseline | 16 | 43.8 ± 22.4 | 19 | 43.9 ± 25.5 | – | – |
| | Post | 13 | 48.3 ± 23.8 | 15 | 39.7 ± 24.3 | -7.34 (-20.63 to 5.95) | 0.279 |
| | 2 weeks | 13 | 48.7 ± 21.5 | 14 | 43.2 ± 22.0 | -5.61 (-19.00 to 7.77) | 0.411 |
| PISQ-12 Total | Baseline | 15 | 31.4 ± 5.0 | 15 | 30.9 ± 9.8 | – | – |
| | Post | 15 | 34.6 ± 5.0 | 11 | 35.6 ± 6.3 | -1.13 (-4.51 to 2.25) | 0.513 |
| | 2 weeks | 15 | 34.4 ± 5.5 | 11 | 33.3 ± 9.3 | -0.88 (-4.26 to 2.49) | 0.609 |
| PSS-10 | Baseline | 20 | 23.5 ± 6.5 | 20 | 24.1 ± 6.5 | – | – |
| | Post | 20 | 23.4 ± 5.2 | 20 | 23.2 ± 5.3 | -0.57 (-3.20 to 2.06) | 0.671 |
| | 2 weeks | 20 | 22.6 ± 6.8 | 18 | 21.7 ± 6.4 | -0.71 (-3.39 to 1.97) | 0.603 |
| SES | Baseline | 20 | 26.9 ± 5.7 | 20 | 26.5 ± 5.2 | – | – |
| | Post | 20 | 26.6 ± 6.5 | 20 | 26.9 ± 6.3 | 0.55 (-1.48 to 2.58) | 0.598 |
| | 2 weeks | 20 | 26.5 ± 6.5 | 18 | 26.9 ± 6.0 | 0.54 (-1.50 to 2.59) | 0.602 |

Data are presented as observed mean ± SD. Adjusted mean differences at post-intervention and 2-week follow-up were estimated using baseline-adjusted linear mixed models with a fixed intercept, fixed effects for group, time, baseline score, and the group × time interaction, and a random intercept for participant. *P* values correspond to adjusted between-group contrasts at each follow-up.

*Abbreviations:* ES, electrostimulation; VR + ES, virtual reality plus electrostimulation; EMG, electromyography; KHQ, King's Health Questionnaire; IIQ-7, Incontinence Impact Questionnaire-7; PISQ-12, Pelvic Organ Prolapse/Urinary Incontinence Sexual Questionnaire-12; PSS-10, Perceived Stress Scale-10; SES, Rosenberg Self-Esteem Scale.

**Supplementary Table S3.** Type III fixed-effect tests from the baseline-adjusted linear mixed models.

| Outcome | | Participants/ observations | Group | Time | Group × Time |
|---|---|---|---|---|---|
| I-QOL | I-QOL total | 36/72 | F(1, 33.00) = 0.49; P = 0.490 | F(1, 34.00) = 0.56; P = 0.460 | F(1, 34.00) = 0.70; P = 0.407 |
| | I-QOL avoidance and limiting behavior | 36/72 | F(1, 33.00) = 1.91; P = 0.176 | F(1, 34.00) = 1.05; P = 0.312 | F(1, 34.00) = 1.81; P = 0.188 |
| | I-QOL psychosocial impact | 36/72 | F(1, 33.00) = 0.04; P = 0.844 | F(1, 34.00) = 1.35; P = 0.254 | F(1, 34.00) < 0.001; P = 1.000 |
| | I-QOL social embarrassment | 36/72 | F(1, 33.00) = 0.48; P = 0.492 | F(1, 34.00) = 0.70; P = 0.409 | F(1, 34.00) = 1.04; P = 0.314 |
| EMG | EMG rest (μV) | 40/80 | F(1, 37.00) = 0.15; P = 0.703 | F(1, 38.00) = 0.07; P = 0.794 | F(1, 38.00) = 0.07; P = 0.794 |
| | EMG work (μV) | 40/80 | F(1, 37.00) = 4.07; P = 0.051 | F(1, 38.00) < 0.001; P = 0.989 | F(1, 38.00) = 0.07; P = 0.799 |
| Bladder-diary outcomes | Mean micturition volume (mL) | 40/80 | F(1, 37.00) = 38.35; P < 0.001 | F(1, 38.00) = 1.18; P = 0.284 | F(1, 38.00) = 3.41; P = 0.072 |
| | Maximum micturition volume (mL) | 40/80 | F(1, 37.00) = 38.88; P < 0.001 | F(1, 38.00) = 0.06; P = 0.812 | F(1, 38.00) = 7.71; P = 0.008 |
| | Minimum micturition volume (mL) | 40/80 | F(1, 37.00) = 2.54; P = 0.120 | F(1, 38.00) = 9.93; P = 0.003 | F(1, 38.00) = 4.16; P = 0.048 |
| | Micturition frequency | 40/80 | F(1, 37.00) = 18.10; P < 0.001 | F(1, 38.00) = 8.87; P = 0.005 | F(1, 38.00) = 0.75; P = 0.393 |
| KHQ | General health perceptions | 31/60 | F(1, 27.99) = 0.56; P = 0.461 | F(1, 27.39) = 6.67; P = 0.015 | F(1, 27.39) < 0.001; P = 0.993 |
| | Incontinence impact | 31/59 | F(1, 28.01) = 0.38; P = 0.542 | F(1, 26.15) = 0.001; P = 0.971 | F(1, 26.15) = 0.001; P = 0.971 |
| | Role limitations | 30/57 | F(1, 27.01) < 0.001; P = 0.999 | F(1, 25.78) = 0.29; P = 0.597 | F(1, 25.78) = 0.77; P = 0.387 |
| | Physical limitations | 30/58 | F(1, 26.85) = 0.005; P = 0.946 | F(1, 25.95) = 1.34; P = 0.257 | F(1, 25.95) = 0.39; P = 0.538 |
| | Social limitations | 30/58 | F(1, 27.01) = 0.08; P = 0.783 | F(1, 26.50) = 0.34; P = 0.563 | F(1, 26.50) = 1.67; P = 0.208 |
| | Personal relationships | 27/52 | F(1, 23.98) = 0.11; P = 0.744 | F(1, 23.15) = 2.29; P = 0.144 | F(1, 23.15) = 0.08; P = 0.785 |
| | Emotions | 30/58 | F(1, 26.99) = 0.001; P = 0.972 | F(1, 26.20) = 0.11; P = 0.741 | F(1, 26.20) = 0.41; P = 0.528 |
| | Sleep/Energy | 31/60 | F(1, 27.99) = 0.51; P = 0.480 | F(1, 27.18) = 1.42; P = 0.243 | F(1, 27.18) < 0.001; P = 0.984 |

| | | | | | |
|---|---|---|---|---|---|
| | Severity measures | 30/56 | F(1, 27.15) = 0.29; P = 0.597 | F(1, 24.30) = 0.05; P = 0.826 | F(1, 24.30) = 0.05; P = 0.826 |
| | IIQ-7 physical activity | 28/55 | F(1, 25.05) = 0.42; P = 0.525 | F(1, 25.14) = 0.20; P = 0.655 | F(1, 25.14) = 0.20; P = 0.661 |
| | IIQ-7 travel | 28/55 | F(1, 25.20) = 1.85; P = 0.186 | F(1, 25.45) = 1.07; P = 0.310 | F(1, 25.45) = 1.71; P = 0.202 |
| IIQ-7 | IIQ-7 social relationships | 28/55 | F(1, 25.29) = 0.51; P = 0.481 | F(1, 25.71) = 0.19; P = 0.667 | F(1, 25.71) = 0.19; P = 0.667 |
| | IIQ-7 emotional health | 28/55 | F(1, 25.28) = 1.87; P = 0.183 | F(1, 25.99) < 0.001; P = 0.997 | F(1, 25.99) = 0.11; P = 0.747 |
| | IIQ-7 total | 28/55 | F(1, 25.14) = 1.05; P = 0.316 | F(1, 25.40) = 0.25; P = 0.622 | F(1, 25.40) = 0.12; P = 0.731 |
| PISQ-12 total | | 28/52 | F(1, 25.39) = 0.40; P = 0.531 | F(1, 23.61) = 0.01; P = 0.911 | F(1, 23.61) = 0.03; P = 0.859 |
| PSS-10 | | 40/78 | F(1, 36.18) = 0.29; P = 0.597 | F(1, 36.02) = 1.91; P = 0.176 | F(1, 36.02) = 0.01; P = 0.911 |
| SES | | 40/78 | F(1, 36.70) = 0.30; P = 0.587 | F(1, 36.00) = 0.26; P = 0.613 | F(1, 36.00) < 0.001; P = 0.998 |

*Note.* All models included a fixed intercept; fixed effects for group, time, the corresponding baseline value, and the group × time interaction; and a participant-specific random intercept. Type III tests used the Satterthwaite approximation for denominator degrees of freedom and sum-to-zero contrasts. Numerator df was 1 for every test. P values were not adjusted for multiple comparisons. Adjusted between-group contrasts and their 95% confidence intervals are reported in Supplementary Table S2.

*Abbreviations*: I-QOL, Incontinence Quality of Life Questionnaire; EMG, electromyography; IIQ-7, Incontinence Impact Questionnaire-7; PISQ-12, Pelvic Organ Prolapse/Urinary Incontinence Sexual Questionnaire-12; PSS-10, Perceived Stress Scale-10; SES, Rosenberg Self-Esteem Scale.
